# Severe Anemia Is Associated with Systemic Inflammation in Young Children Presenting to a Tertiary Hospital in Uganda

**DOI:** 10.4269/ajtmh.20-0199

**Published:** 2020-09-08

**Authors:** Robert O. Opoka, Andrea L. Conroy, Ali Waiswa, Ronald Wasswa, James K. Tumwine, Charles Karamagi, Chandy C. John

**Affiliations:** 1 Department of Paediatrics and Child Health, College of Health Sciences, Makerere University, Kampala, Uganda;; 2 Ryan White Center for Pediatric Infectious Disease and Global Health, Indiana University School of Medicine, Indianapolis, Indiana;; 3 Nalufenya Children’s Ward, Jinja Regional Referral Hospital, Jinja, Uganda;; 4 Global Health Uganda (GHU) Research Collaboration, Kampala, Uganda

## Abstract

The role of inflammation in severe anemia (SA) in African children has not been well characterized. We conducted a study to evaluate risk factors for SA in young children admitted at a tertiary unit in Uganda. Clinical, infectious, and micronutrient risk factors for anemia, along with markers of inflammation, were evaluated in children aged < 5 years in Jinja Hospital, Uganda. Participants included 284 children with SA (Hemoglobin [Hb] < 5.0 g/dL), and two control groups: 63 children admitted with acute illness without SA (Hb > 9.3 g/dL) and 53 asymptomatic community control children. Appropriate logistic analysis was performed to determine factors associated with SA. Of the 284 children with SA, 36.5% had *Plasmodium falciparum* parasitemia, 32.7% had blackwater fever (one of the types of severe malaria), and 15.5% had vitamin B12 deficiency. HIV infection, bacteremia, hookworm infection, severe acute malnutrition, and folate deficiency were relatively uncommon (each accounting for < 8%). Factors independently associated with SA compared with the combined control groups included (adjusted odds ratio [OR]; 95% CI) the following: *P. falciparum* parasitemia (OR: 4.3; 95% CI: 1.4–13.8), total white blood count (OR: 1.3; 95% CI: 1.1–1.4), C-reactive protein (OR: 1.8; 95% CI: 1.3–2.4), and ferritin (OR: 2.7; 95% CI: 1.9–4.0). In this area of Uganda, malaria and markers of inflammation were independently associated with SA in children. Additional studies are required to determine the role of inflammation in children with SA in this population.

## INTRODUCTION

Acute episodes of severe anemia (SA) (Hemoglobin [Hb] ≤ 5 g/dL) are a public health problem among children living in malaria-endemic areas. They account for 9.7–17.8% of hospitalizations, with a case fatality rate of 2.7–13.6%.^1,2^ The causes of SA are multifactorial and interrelated with several of the factors often working together.^3^ However, most studies on SA have evaluated individual etiological factors,^4–7^ and few studies have systematically examined the various etiological factors associated with SA and how these relate to each other. A case–control study of SA in Malawian children identified bacteremia, malaria, hookworm infection, HIV infection, and deficiencies in glucose-6-phosphate dehydrogenase and vitamins A and B12 to be the major contributors of SA.^8^ A similar study conducted in Papua New Guinea^9^ reported that undernutrition, vitamin A deficiency, infection with parvovirus B19, and infection with *Plasmodium falciparum* malaria were the main factors associated with SA. These findings suggest that risk factors for SA depend on specific epidemiological settings and environment.

It is postulated that the various etiological factors lead to SA through one of three pathophysiological pathways: hemolysis, acute or chronic blood loss, and dyserythropoiesis.^10,11^ However, one of the few studies that specifically examined the pathophysiology of acute episodes of SA found that irrespective of the etiology, failure of production of red cells because of bone marrow failure was the common final pathway for SA in that population.^11^ The bone marrow failure was postulated to have been mediated by inflammatory mechanisms with downregulation of erythropoietin production. The authors noted that their findings needed to be confirmed in other settings where etiological factors differ. In Uganda where malarial transmission rates remain high,^12^ the etiological factors associated with SA and the underlying pathological pathways are poorly described. Identification of the etiological factors and pathophysiological pathways involved in SA is necessary to focus context-specific interventions for control and treatment of this common childhood problem.

A number of studies in adults and children show that inflammation plays an important role in anemia, especially in diseases associated with repeated infections, inflammatory disorders, and sepsis.^13,14^ Studies in the critically ill show that inflammation is also an important contributor to anemia of acute illnesses such as severe trauma, myocardial infarction, or sepsis.^15^ In resource-limited settings where infections are common,^16^ inflammatory markers are not well studied as risk factors for SA in children.^17^ Hence, there is need for studies to investigate the role of inflammation alongside the standard clinical, infectious, and micronutrient risk factors for SA in African children.

We evaluated the etiological factors associated with SA in young children presenting to a tertiary unit in Uganda. In addition, we measured erythropoietin and acute inflammatory markers (ferritin and C-reactive protein [CRP]) in the study population to evaluate the association between these biomarkers and SA.

## METHODS AND MATERIALS

### Design.

This was a comparative cross-sectional study carried out at Jinja Regional Referral (Jinja RR) Hospital in the mideastern part of Uganda. Jinja RR Hospital is a free-for-service public facility that mainly serves the rural population from the Busoga region between the shores of lakes Victoria and Kyoga. The Busoga region is an area of high year-round malaria transmission with a parasite prevalence rate of 24–53%.^18^ The pediatric ward at Jinja Hospital is an 80-bed unit that admits 30–50 patients daily. The ward also has a functioning side laboratory able to provide basic microscopy and blood transfusion services.

### Study population.

We consecutively recruited, at admission, children with SA (Hb ≤ 5.0 g/dL) aged 0–5 years presenting to the pediatric ward of Jinja RR Hospital. Two comparative groups of children of similar age were enrolled: 1) a non-SA (Hb > 9.3 g/dL) group of children admitted for acute illnesses without SA, and 2) healthy community children (CC) from the family or neighborhoods of the SA children. For all the groups, children who had been transfused in the last 4 weeks or presenting because of surgical conditions or known chronic conditions such as bleeding disorders, malignancy, or sickle cell anemia were excluded. For the healthy CC, children with febrile illnesses at the time of recruitment were excluded. Recruitment of both SA and comparative groups happened concurrently, and matching was performed at the group and not the individual level. Overall, we aimed to recruit about one child in the comparative groups for every two SA cases that were enrolled.

### Data and sample collection.

A standardized study questionnaire was used to collect data on each child’s sociodemographic details, findings on clinical examination, clinical diagnoses, treatment received, results of routine laboratory investigations, and outcome of the hospitalization. In addition, blood, urine, and stool were collected to investigate for known etiological causes of SA. Serum samples were aliquoted and stored at −80°C for future analysis. Study participants were managed as per Ugandan clinical guidelines.^19^ Children with SA received a blood transfusion, if indicated, followed by specific treatment for any comorbid condition identified. All children with evidence of malaria (positive rapid diagnostic test [RDT] or parasites detected by microscopy) were given parenteral artesunate and/or artemether–lumefantrine as per Ugandan guidelines.

### Measurements.

Height (or length in children younger than 2 years) was measured after recovery using a portable stadiometer, and weight was measured using a digital weighing scale (SECA, Hamburg, Germany). Hemoglobin concentration was measured on-site with a HemoCue system (HemoCue 301, Angelholm, Sweden). The Hb measurement by HemoCue was used to determine eligibility for enrollment and subsequent study group. A complete blood count with differentials was performed using a hematological analyzer at Lancet Laboratories (Beckman Coulter, Brea, CA).

All children had a routine blood smear (Field’s stain) and three band (*P. falciparum*/Pan) rapid diagnostic malaria test (SD Bioline Malaria Ag *P. falciparum*/Pan, Abbott, Chicago, IL) at admission. In addition, Giemsa-stained thick and thin smears were prepared and read independently by two certified malaria microscopists. *Plasmodium falciparum* asexual parasites were counted against 200 or 500 white blood cells, and parasite density was calculated based on an estimated total white blood cell count of 8,000/µL. Reticulocyte counts were also read manually on new methylene blue N stained slides by two microscopists. For both malaria and reticulocyte counts, a third reader was required in the event of discordant results or a parasite density or reticulocyte counts that differed by more than 20%. The reticulocyte percent was calculated out of the number of reticulocytes counted in 1,000 red blood cells.

For stool and urine samples, caregivers were provided with clean stool and urine containers to collect a single early morning sample from the study participants. Stool samples were examined for helminths within 1 hour of collection using direct microscopy and iodine preparation. A wet preparation was performed on the fresh urine sample and examined by direct microscopy for *Schistosoma haematobium*. The microscopy for urine and stool was performed from the side laboratory of the hospital.

Blood culture: Venous blood samples of 1–2 mL were taken for all children with axillary temperature > 38.0°C. Samples were incubated in BACTEC 9050 (BD, Franklin Lakes, NJ) automated system for at least 5 days. Mixed growths or coagulase-negative *Staphylococcus* were considered contaminants.

HIV testing was performed for all children using the rapid diagnostic screening tests (Determine HIV1/2, Abbott, Chicago, IL; HIV1/2 Stat-Pak, Chembio, Medford, NY, and Uni-Gold, Trinity Biotech, Bray, Ireland). Children who were younger than 18 months had their positive HIV screening result confirmed by DNA PCR.

To check for sickle cell genes, Hb electrophoresis was performed on dried blood spots from the Central Public Health Laboratories in Kampala.

### Assays for biomarkers and micronutrients.

Laboratory assays were performed on stored serum sample aliquots.

#### Assessment of vitamin B12 and folic acid.

Vitamin B12 and folic acid were measured in serum using microbiological assays (Eagle Biosciences, Amherst, NH). For vitamin B12 assessment, samples (diluted 1:25) and standards were added to microtiter plates coated with *Lactobacillus delbrueckii* ssp. *lactis* and incubated at 37°C for 44–48 hours. The amount of vitamin B12 was determined based on the amount of growth of *Lactobacillus plantarum* measured using turbidimetry at 610 nm using a SpectraMax M3 multi-mode spectrophotometer (Molecular Devices, San Jose, CA). We tested 10% of samples in duplicate and had a coefficient of variation of 15.6% for vitamin B12. The assay range was 2700–75 ng/L. For folic acid, a similar protocol was used, but samples were diluted 1:75 and plated on a microtiter plate coated with *Lactobacillus rhamnosus.* The assay range was 48–1.5 µg/L, and the coefficient of variation for samples tested in duplicate was 3.3%.

#### Assessment of CRP and ferritin.

C-reactive protein (DuoSet ELISA, R&D Systems, Minneapolis, MN) and ferritin (Ramco Laboratories, Stafford, TX) were measured in serum by ELISA according to the manufacturer instructions. Dilutions for CRP were 10,000, 200,000, or 250,000 and ferritin were 10, 2, or neat (undiluted). The assay coefficient of variation for CRP and ferritin was 5.3% and 1.9%, respectively. The assay range for CRP was 0.115–750 mg/L and for ferritin was 3–40,000 ng/mL.

### Definitions.

Severe malaria was defined as the presence of asexual forms of *P. falciparum* by microscopy. Recent malarial infection was defined as positive RDT result in the absence of parasitemia on microscopy. Blackwater fever was defined as a history of passage of dark brown, tea, or cola-colored urine during the present episode of illness observed by the caregiver and where possible by the study clinician. To ensure accuracy, color schemes were used to confirm the color of the urine.

Calculations for anthropometric *z*-scores were performed according to WHO growth reference curves with the use of Epi Info 2000 (CDC, Atlanta, GA). Severe acute malnutrition was diagnosed as weight-for-height *z*-scores less than −3 SD or between −2 and −3 in the presence of significant edema. Stunting was defined as a height-for-age *z*-score of less than −2.

Vitamin B12 and folate deficiencies were considered present if concentrations were < 200 pg/mL and 3 ng/mL, respectively.^20–22^ Iron deficiency was defined as ferritin < 12 µg/L if CRP was < 10 mg/L or ferritin < 30 µg/mL if CRP was ≥ 10 mg/L.^23^ Limited maternal education was defined as having spent less than 7 years at school or having no education.

### Sample size.

We estimated that a sample of 399 children at a ratio of 2:1 between the SA and comparator groups would give us 80% power to detect a 15% difference in proportion of children with malaria between the two groups, at 0.05 level of significance.

### Statistical analysis.

Data were entered in FileMaker Pro and exported to Stata version 14 for analysis (StataCorp., College Station, TX). Descriptive statistics were used to present the data and to determine differences in the study groups. For comparisons, the chi-squared test or Fisher’s exact test was used for categorical data, whereas for continuous data, means were compared using the Student’s *t*-test and medians using the Wilcoxon rank-sum test. Among the SA children, we determined the frequency of each of the known etiological factors and also determined for each patient the number of associated etiological factors identified. Bivariate logistic regression was used to determine factors associated with SA. In addition to factors known to be associated with SA, that is, age, gender, and maternal education, we also included in the multivariable model factors that were significant (*P* < 0.05) at bivariate analysis. Recent malaria exposure, severe acute malnutrition, and blackwater fever were excluded from the final multivariable model because they were collinear with other variables in the model.

### Ethical considerations.

Ethical approval was provided by the Makerere University School of Medicine Research and Ethics Committee and the Uganda National Council of Science and Technology. Written informed consent was obtained from the caregivers of all the children.

## RESULTS

Between June 2016 and January 2018, a total of 417 children were screened and recruited. Seventeen SA children were excluded, one with Hb > 5.0 g/dL and 16 with sickle cell anemia. Four hundred children (SA, *n* = 284; non-SA, *n* = 63; and CC, *n* = 53) were eligible and were included in the analysis. The mean Hb for SA, non-SA, and CC was 3.6, 11.1, and 11.3 g/dL, respectively (Table 1). There was no difference in the mean age across the three groups. Among the hospitalized children, fever and cough were the most common presenting symptoms (Table 1). Severe anemia children were more likely to be referred, febrile at admission, and had longer mean duration of hospitalization than non–SA children (Table 1). Among the SA children, 153 (53.9%) had been previously hospitalized with 104 (67.9%) of prior hospitalizations requiring a blood transfusion (Table 1). There were two inpatient deaths in the study, both in the SA group.

**Table 1:** Demographics, clinical characteristics, and inpatient outcome of study participants

	SA (*N* = 284)	Acute illness, no SA (*N* = 63)	Asymptomatic community children (*N* = 53)
Age (years), mean (SD)	2.3 (1.2)	2.1 (1.2)	2.3 (1.0)
Gender, male, *n* (%)	179 (63.0)	29 (46.0)	32 (60.4)
Stunted, *n* (%)*	74/276 (26.8)	12 (19.1)	19 (35.9)
Limited maternal education†	217 (78.1)	29 (46.0)	35 (71.4)
Axillary temperature (°C), mean (SD)	37.4 (0.8)	36.9 (0.9)	36.5 (0.4)
Referred, *n* (%)	172 (60.6)	5 (7.9)	–
Immunized up to date, *n* (%)	274 (96.5)	61 (96.8)	–
Presenting features, *n* (%)			
Fever	280 (98.6)	44 (69.8)	–
Cough	200 (70.4)	46 (73.0)	
Duration of illness (days), mean (SD)	3.9 (1.8)	3.1 (2.4)	–
Previous history of, *n* (%)			
Hospitalization	153 (53.9)	20 (31.8)	–
Transfusion	104 (67.9)	0 (0.0)	
Transfused > 1	57 (54.8)	0 (0.0)	
Clinical presentation, *n* (%)			
Febrile (*T* > 37.4°C)	132 (46.5)	16 (25.4)	–
Jaundice	62 (21.8)	0 (0.0)	
Hepatomegaly	71 (25.0)	0 (0.0)	
Splenomegaly	159 (55.9)	0 (0.0)	
Duration of hospitalization, mean (SD)	4.4 (2.1)	1.5 (1.9)	–
Hemoglobin level (g/dL), mean (SD)	3.6 (0.9)	11.1 (1.1)	11.3 (1.6)
Total WBC, mean (SD)‡	19.3 (13.2)	9.3 (3.3)	10.2 (3.2)
Reticulocytosis (> 2.5%), *n* (%)	50 (17.6)	12 (19.1)	7/52 (13.5)
Died, *n* (%)	2 (0.7)	0 (0.0)	–

SA = severe anemia.

* Stunted = height-for-age *z*-score < −2 SD.

† Data available for SA (*n* = 278) and non-SA (*n* = 49).

‡ WBC = white blood count, data available for SA, *n* = 280; non-SA, *n* = 50; CC, *n* = 51.

### Factors associated with SA.

*Plasmodium falciparum* malaria and blackwater fever were the most common diagnoses among SA children, accounting for 103 (36.5%) and 93 (32.7%), respectively (Table 2). A total of 143 (50.4%) SA children had a recent malaria infection defined as a positive RDT for HRP2 and negative blood slide at the time of admission (Table 2). Asymptomatic parasitemia was found in 5 (9.4%) of the CC, whereas 5 (7.9%) of non–SA children had malaria (Table 2). Severe anemia children had higher mean white blood cell counts than non-SA or CC (Table 2). In addition, the levels of markers of acute inflammation (serum ferritin and CRP) were significantly higher in the SA children than in children in the control groups (Table 2). There was, however, no statistically significant difference in the proportion of children with high reticulocyte count in the three groups (Table 2).

**Table 2:** Risk factors for SA in children with SA compared with control groups

Risk factors, *n* (%)	SA (*N* = 284)	Control groups		*P*-value
		Non-SA (*N* = 63)	SA vs. controls*	
Clinical				
Recent malarial infection	143 (50.4)	10 (15.9)	17 (32.1)	< 0.001
Severe acute malnutrition	19 (6.7)	0 (0.0)	0 (0.0)	0.004
Infection or infection-related				
Malaria parasitemia	103 (36.5)	5 (7.9)	5 (9.4)	< 0.001
Blackwater fever	93 (32.7)	1 (1.6)	0/53 (0)	< 0.001
Hookworm infection	16/222 (7.2)	1/50 (2.0)	2/21 (9.5)	0.374
HIV infection	7 (2.5)	2 (3.2)	0 (0)	0.650
Bacteremia	3/54 (5.6)	0/9 (0.0)	–	0.469
Micronutrient deficiency				
Vitamin B12 deficiency	44 (15.5)	6 (9.5)	–	0.222
Folate deficiency	5 (1.8)	0 (0.0)	–	0.289
Markers of inflammation				
C-reactive protein† (mg/L), mean (SD)	5.4 (1.0)	2.8 (2.4)	0.2 (1.8)	< 0.001
Ferritin level‡ (mg/L), mean (SD)	8.1 (1.7)	4.9 (1.6)	4.1 (1.4)	< 0.001

CC = community children; SA = severe anemia.

* Control = non-SA and CC combined.

† Data available for SA, *n* = 277; log values presented.

‡ Data available for SA, *n* = 278; log values presented.

#### Micronutrient deficiencies.

There was no difference in the prevalence of vitamin B12 and folate deficiencies between children with SA and those in the control groups. Iron deficiency could not be accurately measured because many children had very elevated ferritin and CRP levels, suggesting inflammation that altered ability to use ferritin as a marker of iron deficiency.

#### Infections.

The prevalence of HIV infection was 7 (2.7%) in SA, 2 (3.2%) in non-SA, and 0/53 (0.0%) in CC (Table 2). A blood culture was performed in a total of 54 SA children, of which 3 (5.6%) were positive. The invasive bacteria species identified were *Citrobacter*, *Providencia*, and *Staphylococcus aureus*. Stool samples were available for 293 (73.3%) study participants, and hookworm ova were seen in 16 (7.2%) children with SA. The prevalence of hookworm infection was not different between children with SA and the control groups (Table 2). Urine samples were collected from 287 participants, including 234 (77.7%) children with SA. However, none of the samples were positive for *S. haematobium*.

Overall, nearly half of the children had an associated etiological factor identified for SA (*n* = 128, 45.1%), whereas 69 (24.3%) children had multiple associated etiological factors identified. The majority (70/87; 80.1%) of the SA children with no associated etiological factors identified had recent malarial infection.

#### Regression analysis of independent factors associated with SA.

To determine the etiological factors and biomarkers associated with SA, we used logistic regression. The final model compared the factors between children with SA and those in the control groups (non-SA and CC) combined. We found the factors associated with SA were malaria parasitemia (OR: 4.3; 95% CI: 1.4–13.8), unit increase in total white blood count (OR: 1.3; 95% CI: 1.1–1.4), CRP (OR: 1.8; 95% CI: 1.3–2.4), and ferritin (OR: 2.7; 95% CI: 1.9–4.0) (Table 3). These findings were similar even when children with SA were compared separately with either of the control groups (non-SA or CC).

**Table 3:** Etiological factors associated with severe anemia in children younger than 5 years presenting to Jinja Regional Referral Hospital

	Unadjusted		Adjusted*	
	OR (95% CI)	*P*-value	OR (95% CI)	*P*-value
Malaria parasitemia	6.0 (3.0, 12.0)	< 0.001	4.4 (1.4, 13.8)	0.012
Total white blood count	1.3 (1.2, 1.3)	< 0.001	1.3 (1.1, 1.4)	< 0.001
C-reactive proteins (mg/L)	2.9 (2.3, 3.6)	< 0.001	1.8 (1.3, 2.4)	< 0.001
Ferritin (mg/L)	4.1 (3.0, 5.6)	< 0.001	2.7 (1.9, 4.0)	< 0.001
Age	1.1 (0.95, 1.4)	0.170	–	–
Gender	0.65 (0.4, 1.0)	0.054	–	–
Vitamin B12 deficiency	1.7 (0.7, 4.3)	0.227	–	–
Hookworm infection	3.8 (0.5, 29.4)	0.200	–	–
HIV infection	1.6 (0.3, 7.0)	0.652	–	–

* Also adjusted for age, gender, and maternal education.

## DISCUSSION

We evaluated the etiological factors and biomarkers of host response in hospitalized children with SA in Jinja RR hospital. We identified multiple etiological factors associated with SA, which is consistent with results from other studies in malaria-endemic areas.^8,9,24^ In this study, like others in malaria-endemic areas, *P. falciparum* was an important contributor to SA in young children. The prevalence of malaria parasitemia was 36.5% in children with SA and was lower than the 54% reported in earlier studies from similar settings.^1^ In this study, we found that SA children had markedly elevated levels of ferritin and CRP (markers of host response) compared with control children.

Malaria remains a significant contributor to SA. In this study, an additional 50.5% of the children with SA, although malaria smear negative by microscopy, had recent exposure to malaria based on a positive RDT test. This suggests that malaria exposure in the community is still high despite the ongoing malaria control interventions being implemented.^25^ The high rate of RDT-positive but smear-negative malaria may be due to the availability and easy access to effective antimalarials in the community that rapidly clear parasitemia before presentation to hospital. It is also probable that some of the RDT-positive malaria was due to submicroscopic parasitemia, a phenomenon that is increasingly being recognized in malaria-endemic areas.^26,27^ Irrespective of the reasons for the negative smear, RDT-positive malaria is likely a significant contributor to the high burden of SA found in this community. It is possible that these smear-negative children present before making a complete recovery from malaria-related anemia of the recent malarial episode. It is also possible that the anemia may be due to post-artesunate delayed hemolysis that has been reported in some children.^28–30^ Taken together, our findings support the assertion in malarial-endemic areas that it is exposure rather than the level of parasitemia that is important in the development of SA.^26,31,32^ Efforts to reduce SA in children living in malaria-endemic areas should therefore focus on preventing exposure to malaria.

Blackwater fever, a form of severe malaria, was one of the most prevalent etiological factors of SA identified in this study. Blackwater fever is common in eastern Uganda and presents in up to 21% of critically ill hospitalized children.^33,34^ Children who develop this condition are prone to recurrent episodes of SA requiring repeated blood transfusions. The etiology of blackwater fever and trigger for SA in this condition are not known. In this study, the prevalence of blackwater fever was similar in children with recent malarial infections and those with smear-positive malaria. So, it is unlikely to be a marker of recent malarial infection. Blackwater fever was also not an independent predictor of SA as it was correlated to malaria parasitemia. It is however possible that blackwater fever may be a marker of more significant hemolysis that might be genetic or related to parasitemia. Further research is needed to establish the pathophysiology and treatment of this condition.

Other etiological factors were relatively uncommon in this study. The HIV prevalence rate in the study was lower than the 12.6% reported in the Malawian study^8^ but similar to recent HIV rates reported in hospitalized children in East Africa.^33,35^ The prevalence of bacteremia in this study was also lower than that in the Malawian study where 15% of SA children were culture positive,^8^ but similar to what has been reported in malarial studies in similar settings.^9,35^ An earlier study conducted at Jinja RR Hospital reported a bacteremia rate of 19% among febrile young children without parasitemia.^36^ However, that study was conducted before the introduction of pneumococcal conjugate vaccine for routine childhood immunization in Uganda. There is presently an ongoing surveillance study to determine the impact of this new vaccination on childhood inpatient morbidity.

The prevalence of undernutrition, and folate and vitamin B12 deficiencies was relatively low in the study population and did not differ between groups. We were unable to make conclusions on the level of iron deficiency in this study because of the high level of inflammation found in the study patients. The role of iron deficiency in the etiology of SA however remains unclear, with some studies finding it protective^8^ and others not protective,^9^ whereas in others, it was a significant risk factor for SA.^24^ The Papua New Guinea study also found that vitamin A deficiency and hookworm infection were associated with SA,^9^ although hookworm infection was not associated with SA in this study. The relatively low prevalence of hookworm infection is likely attributable to the national deworming program in Uganda where all children younger than 5 years are dewormed every 6 months.

In this study, we found that SA was associated with the biomarkers of host response as seen in the markedly raised levels of CRP and ferritin. The high levels of these biomarkers of host response found in this study support findings from previous studies of the central role that inflammation plays in the pathogenesis of SA, irrespective of the etiology, in malaria-endemic areas.^37^ However, the pathogenic pathways responsible for the development of SA remain unclear. Studies on the pathogenesis of severe malarial anemia suggest that anemia mainly results from the suppression of erythropoietin-induced erythropoiesis by parasite factors, such as hemozoin,^38^ mediated by inflammatory cytokines.^10^ In Malawian children, it was found that irrespective of the etiological factor, suppression of erythropoiesis leading to red cell production failure was the predominant pathway for the development of SA.^11^ Interestingly, we found no significant difference in reticulocyte count across groups. This suggests a failure of the bone marrow in the SA group, given the profound anemia this group. It is likely that insufficient iron delivery to the bone marrow to support new red cell formation happens in the context of anemia of inflammation with iron sequestration and decreased iron uptake in the gut due to hepcidin binding and degrading gut ferroportin. Overall, our finding of the strong association between the markers of inflammation and SA highlights the central role of inflammatory mechanisms in the pathways leading to SA.

The levels of ferritin and CRP differed between the different forms of SA children. Ferritin levels were highest in SA children without malaria, whereas CRP was highest in the group with parasitemia and lowest in SA children without malarial infection. Taken together, these findings suggest that the inflammatory markers involved and the mechanisms of suppression on red cell production may be different for malarial and non-malarial causes of SA. Further research is needed to understand the mechanisms by which inflammation mediates dyserythropoiesis for the various etiologies of SA.

Severe anemia children also had significantly higher mean total white blood cell counts than children in the control groups. Among SA children, the levels of white blood cell counts were significantly elevated in children with recent malarial infection and no malaria than in children with parasitemia. This may be due to the strong association between recent malaria exposure and increased risk of Gram-negative bacteremia.^39,40^ Although the prevalence of bacteremia was low in this study, the elevated levels of white blood cell count may be markers of infection in children without parasitemia. Children with recent malarial infection had higher (8.7%) levels of bacteremia than those with parasitemia (3.7%), although the difference was not statistically significant.

The inpatient mortality in this study was relatively low, confirming findings that with SA, prompt availability of blood transfusion services is the critical factor in inpatient mortality.^41^ However, more than a third (36.7%) of the SA children had a history of previous transfusion, suggesting that SA is a recurrent problem in these children. Thus, there is need for longitudinal studies to identify SA children at risk of poor post-discharge outcomes.

Our study had some limitations. We were not able to evaluate for all possible known causes of SA. For example, we did not do viral or genetic studies, so possible viral and genetic factors were not documented. The biomarkers of host response were measured once, and so we were not able to establish the temporal relationship with the development of SA. In addition, we were unable to do bone marrow biopsies to establish the functionality of the bone marrow for the various etiologies of SA. However, we evaluated for the most common available causes of SA in our setting.

In summary, we identified multiple etiological factors associated with SA in young children presenting to Jinja Hospital. *Plasmodium falciparum* malaria and blackwater fever were the most prevalent etiological diagnoses in SA children. In multivariable analysis, markers of host responses, that is, ferritin and CRP, were significantly associated with SA. There is need for studies to confirm the immunopathogenic pathways of SA in malaria-endemic areas to target intervention strategies.
